# Some Additional Concepts for Encouraging Trainees to Academic Writing

**DOI:** 10.31662/jmaj.2024-0008

**Published:** 2024-08-09

**Authors:** Shigeki Matsubara

**Affiliations:** 1 Department of Obstetrics and Gynecology, Jichi Medical University, Tochigi, Japan; 2 Department of Obstetrics and Gynecology, Koga Red Cross Hospital, Koga, Japan; 3 Medical Examination Center, Ibaraki Western Medical Center, Chikusei, Japan

**Keywords:** academic writing, paper, paper writing, review, trainee

## Dear Editors,

I thank Dr. Saeki for thoughtfully considering our comments ^(1)^. He highlighted the significance of motivating trainees to contribute to academic literature, especially through case reports ^(2)^. I stressed that case reports should not be exclusively assigned to trainees; rather, senior physicians can and should write when trainees are unavailable ^(3)^. I believe Saeki fundamentally concurred with this perspective, offering additional recommendations worth consideration ^(1)^. To conclude our exchange of ideas ^(1), (2), (3)^, I wish to make three additions.

First, medical papers fundamentally serve the interests of societies and patients, not the authors or trainees. The most undesirable scenario arises when attending physicians, typically younger ones, either fail to write or produce inaccurate reports, leading to the burying or misinterpretation of valuable data. In such cases, senior physicians must intervene and take charge ^(2)^. This is because, irrespective of the author, a paper must effectively convey its message. It is the responsibility of senior physician. Writing papers is akin to on-the-job training and comparable to surgery. The senior surgeon, not the trainee, bears the greatest responsibility for the outcome. If a trainee is expected to perform a procedure incorrectly, the senior surgeon must intervene immediately.

Second, there are various ways to involve trainees in the paper review process. Many journals welcome the participation of younger doctors. When engaging trainees, please include their names on the review sheet, as many journals provide a designated space to do so. Alternatively, you can recommend your trainees to journals and ask for formal invitations to have them serve as reviewers.

Third, I would like to share my experiences. Seasoned professionals in academic writing “outside” can support trainees. In 2010, I established the Clinical Research Support Team (CRST) ^(4), (5)^ to aid younger doctors in writing. Younger doctors associated with Jichi Medical University (JMU), particularly JMU graduate doctors in rural areas, can email the CRST for assistance in preparing a paper. The CRST comprises approximately 230 doctors, primarily JMU senior staff and provides support until the paper is accepted. About 100 papers have been published with the assistance of CRST.

I echo Dr. Saeki’s perspective on the importance of educating younger audience in academic writing. By voluntarily establishing and sustaining CRST, I will continue to contribute to nurturing the writing skills of younger generations. I aim to share insights with readers on effectively involving younger doctors in academic writing, with a constant awareness that papers can serve the interests of society and patients.

## Article Information

### Conflicts of Interest

None

### Acknowledgement

This is a personal view and does not represent the opinion of any departments or medical societies with which I am associated.

### Author Contributions

S. Matsubara: Identification of the significance and manuscript writing.

### Approval by Institutional Review Board (IRB)

Not applicable

### Data Availability

Data sharing is not applicable to this article, as no new data were created or analyzed in this study.

### Patient Anonymity

Not applicable

### Informed Consent

Not applicable
